# Distribution and diversity of cyanobacteria in the Azores Archipelago: An annotated checklist

**DOI:** 10.3897/BDJ.10.e87638

**Published:** 2022-09-02

**Authors:** Rúben Luz, Rita Cordeiro, Amélia Fonseca, Pedro Miguel Raposeiro, Vítor Gonçalves

**Affiliations:** 1 CIBIO, Centro de Investigação em Biodiversidade e Recursos Genéticos, InBIO Laboratório Associado, Pólo dos Açores, Ponta Delgada, Portugal CIBIO, Centro de Investigação em Biodiversidade e Recursos Genéticos, InBIO Laboratório Associado, Pólo dos Açores Ponta Delgada Portugal; 2 Faculdade de Ciências e Tecnologia, Universidade dos Açores, Ponta Delgada, Portugal Faculdade de Ciências e Tecnologia, Universidade dos Açores Ponta Delgada Portugal

**Keywords:** Oceanic islands, biodiversity, Cyanophyceae, Macaronesia, Atlantic Ocean

## Abstract

**Background:**

Knowledge about cyanobacteria diversity in the Azores is spread over several publications, dating from 1874, with some of them not generally available to the scientific community due to their restricted access. The dispersion and sometimes inaccessibility of this information hinder a deeper analysis and a better understanding of the biodiversity of the Azores Islands and more general ecological processes in oceanic islands. Here we present the first checklist of cyanobacteria for the Azores Archipelago with updated taxonomy of all recorded taxa.

**New information:**

This work provides a compiled and annotated checklist of all known cyanobacteria from the Azores Archipelago with morphological identification from preserved samples and cultures, based on published literature. All records of taxa known to occur in the Azores were taxonomically updated. The present checklist comprises 225 taxa distributed by six orders (Chroococcales, Nostocales, Oscillatoriales, Pleurocapsales, Spirulinales and Synechococcales). Our literature review reveals that the Azores Archipelago hosts a high diversity of cyanobacteria, despite several overlooked habitats that may present great potential regarding cyanobacteria diversity. Increasing efforts to study these neglected habitats could contribute to the knowledge of cyanobacteria taxonomy. This checklist provides the basis for future works on the taxonomy and taxa richness of cyanobacteria in the Azores and the Atlantic Islands, as also for understanding and monitoring non-indigenous and invasive species.

## Introduction

Cyanobacteria are gram-negative photosynthetic prokaryotes that developed around 3500 million years ago (Schirrmeister et al. 2015). As one of the most primitive organisms on earth (Mareš et al. 2013), they successfully occupy various habitats in terrestrial and aquatic ecosystems, both marine and freshwater (Whitton and Potts 2012). Cyanobacteria diversity amongst these systems is unbalanced, being larger in freshwater and terrestrial ecosystems (Komárek and Johansen 2015). They persist in almost all types of illuminated habitats, with optimum growing temperatures generally higher than microalgae, which enable them to support a wide array of stress conditions, including extreme habitats (Komárek and Johansen 2015).

Freshwater cyanobacteria are commonly present in wetlands, lakes, rivers and streams, both in benthic (Scott and Marcarelli 2012) and planktonic (e.g. Stockner et al. 2000, Oliver et al. 2012) habitats. Benthic cyanobacteria are commonly found solitary or forming mats in the various stream and river substrates, such as rocks, sand, plants and many others (Casamatta and Hašler 2016). In shallow lakes and littoral zones of deep lakes, benthic species of cyanobacteria can also occur if enough light reaches the substrates (Scott and Marcarelli 2012). However, cyanobacteria are mostly known from the plankton of lentic waters, where they can grow in high abundance, usually known as blooms, especially in eutrophic lakes (Paerl et al. 2010, Carmichael and Boyer 2016). Cyanobacteria blooms negatively affect the ecosystems and services they provide (Carmichael and Boyer 2016) as most bloom-forming species produce toxins that can be accumulated at the water surface, causing unpleasant surface scums.

Extreme habitats, such as thermal springs, are successfully occupied by Cyanobacteria, where they are often the main and/or sole autotrophic organisms inhabiting these environments (Komárek and Johansen 2015). In marine systems, cyanobacteria are found in a wide array of habitats, including benthos, plankton, associated with other organisms, amongst others (Golubic et al. 2010, Konstantinou et al. 2018).

In the last ten years, cyanobacteria taxonomy has changed dramatically with the use of new techniques, mainly through 16S rRNA sequencing, contributing to a taxonomic re-assessment of the group (e.g. Wacklin et al. 2009, Komárek et al. 2011, Zapomělová et al. 2011, Strunecký et al. 2013, Komárek et al. 2014, Strunecky et al. 2017, Mai et al. 2018).

In the Azores, a remote oceanic archipelago located in the middle of the North Atlantic Ocean, the first work to be published on cyanobacteria taxonomy came from the Challenger expedition that occurred from 1872 to 1876 and had a brief passage in São Miguel Island from 3 July to 9 July 1873 (Brock and Brock 1967). Some members of the Challenger expedition visited the Furnas Village and later Moseley (1874) and Archer (1874), who received samples from Moseley, published the first records. Later, Trelease (1897) and Bohlin (1901) contributed with several cyanobacteria records from several islands. In the 20^th^ century, more biologists visited the Islands contributing considerably to the knowledge of the microalgae and cyanobacteria of the Azores. First by Krieger (1931), with a small contribution and after with the valuable works of Cedercreutz (1941), Bourrelly and Manguin (1946) and Johansson (1977), contributing with several detailed descriptions of the cyanobacteria flora in several islands of the Azores. The later works contributed with the highest number of known species for the Azores. Bourrelly and Manguin (1946) also describes a new form *Oscillatoriageitlerif.major* Bourrelly in Bourrelly and Manguin (1946), which is the first and only known endemic cyanobacteria to the Azores. After 1980, works were mainly focused on planktonic freshwater species due to the rise of lake eutrophication signs. Important contributions to the known cyanobacteria flora have been provided after the implementation of the Water Framework Directive (WFD) in the Azores, with regular monitoring programmes since 1994 (Santos et al. 2005, Santos et al. 2012, Luz et al. 2020b). The more recent works on cyanobacteria were based on cultured strains isolated from freshwater lakes (Cordeiro 2015), which provide the addition of new species. Several works performed on thermal, terrestrial, brackish and marine habitats, which were overlooked in previous studies, contributed to several new cyanobacteria species records (Luz 2018, Cordeiro et al. 2020b).

Despite the increased research efforts, especially in the last decade, the knowledge of the diversity and distribution of Cyanobacteria in the Azores Archipelago is not consistently organised and a local checklist has never been published. This study aims to present an updated checklist of cyanobacteria present in the Azores, based on a taxonomically updated list of previously reported species from preserved samples and based on cultured strains.

## Materials and methods

### Study Area

**Figure 1.**
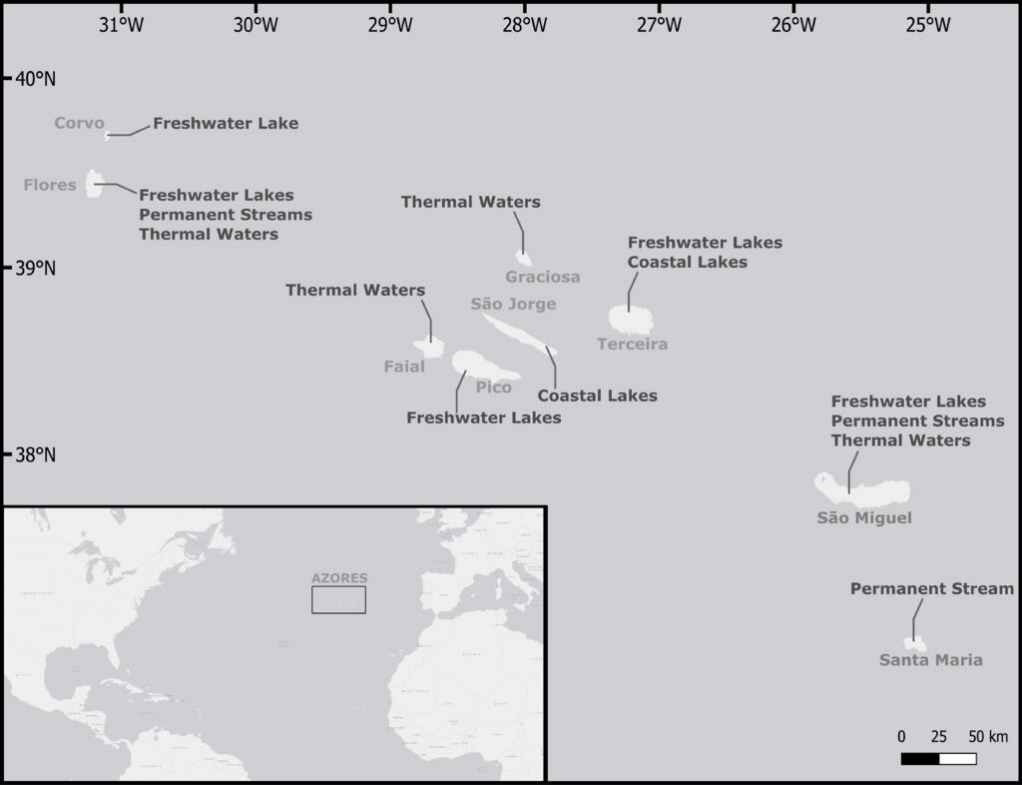
Azores Archipelago location with an indication of the most represented aquatic habitats on each island.

The Azores are an oceanic group of islands located in the middle of the North Atlantic Ocean, roughly 1500 km from Europe and 1900 km from America (Fig. 1). The Archipelago is made up of nine islands roughly aligned along 615 km in a WNW-ESE trend, that are divided into three groups according to their geographical position. Although they are in geographical proximity, the Islands present unique features differentiating themselves from each other (Table 1), with different amounts of annual rainfall (Secretaria Regional do Ambiente e do Mar 2011) and distinct geological settings (e.g. Moore 1990, Azevedo and Portugal Ferreira 2006, Cole et al. 2008).

The western group includes the Islands of Flores and Corvo, which are amongst the smallest islands of the Archipelago. Corvo and Flores are very rich in aquatic habitats despite their small size due to their higher annual precipitation (Secretaria Regional do Ambiente e do Mar 2011). The central group (Graciosa, Faial, Pico, São Jorge and Terceira Islands) comprise the youngest in the Archipelago (Ávila et al. 2016). The islands in the central group include a high diversity of inland aquatic habitats, including freshwater and saline lakes, streams and thermal waters (Morton et al. 1997, Porteiro 2000, Cruz and França 2006, Morton 2014). The eastern group, Santa Maria and São Miguel, includes the Archipelago’s oldest (Santa Maria) and the largest (São Miguel) islands. São Miguel is the island with most of the lakes and the larger area of water bodies (Porteiro 2000), whereas Santa Maria is the driest island of the Archipelago, with only 775.2 mm mean annual precipitation and no significant inland water habitats (Secretaria Regional do Ambiente e do Mar 2011).

The Azores are particularly rich in freshwater systems, with 88 lakes (Porteiro 2000), nine permanent streams, five saline lakes and several thermal springs (Table 1). Lakes are located between 230 and 1,050 m altitude and, according to Gonçalves 2008, could be classified into two main lake types: shallow lakes, with a maximum depth below five metres; and (ii) deep lakes, with maximum depths, greater than five metres. The insular lotic systems are small, narrow, with steep watersheds and are fed by lakes or springs, most of them having torrential or seasonal flowing regimes (Raposeiro et al. 2013).

### Checklist Production

The checklist was based on all known literature mentioning cyanobacteria from the Azores with morphological identification, published until 2020. The nomenclature was revised according to Guiry and Guiry (2022). The complete taxonomic list (taxon data table and occurrence data table) is published in DwC (Suppl. material 1) in GBIF, the Global Biodiversity Information Facility (Luz et al. 2022). Taxa identified only to the family level or above were not included in the discussed taxonomic list.

## Data resources

### Cyanobacteria occurrence in the Azores

**Data set name**: Cyanobacteria Checklist of the Azores Archipelago, Portugal - Occurrence data table

**Data format**: Darwin Core

**Description**: Cyanobacteria occurrence records in the Azores Archipelago, dating from 1874 to 2020, with 2838 records (Luz et al. 2022). Used Darwin Core terms are described in Table 2.

### Cyanobacteria checklist from the occurrence

**Data set name**: Cyanobacteria Checklist of the Azores Archipelago, Portugal - Taxon data table

**Data format**: Darwin Core

**Description**: Cyanobacteria taxa recorded in the Azores Archipelago, based on the occurrrence data table, with a total of 229 taxa (Luz et al. 2022). Used Darwin Core terms are described in Table 3.

## Checklists

### Cyanobacteria checklist from Azores islands

#### Anabaena

Bory ex Bornet & Flahault, 1886

AD225C70-E126-51B1-92A0-EB32FCA1E43B

##### Distribution

Corvo (INOVA 1996), Flores (Bourrelly and Manguin 1946), Pico (Luz et al. 2020), São Miguel (Bohlin 1901), Terceira (Luz et al. 2020)

##### Notes

Freshwater (lake), thermal (pool)

#### Anabaena aspera

Frémy, 1930

0D2AD3D2-D028-58BE-A590-48FA64D17004

##### Distribution

Flores (Bourrelly and Manguin 1946)

##### Notes

Freshwater (lake)

#### Anabaena augstumalis

Schmidle, 1900

A08E012F-C9DA-5AF1-BB70-C235C1D41E16

##### Distribution

Flores (Bourrelly and Manguin 1946)

##### Notes

Freshwater (lake)

#### Anabaena cylindrica

Lemmermann, 1896

4111C797-D7D8-5993-AFA8-0D5E4BD049C8

##### Distribution

São Miguel (Oliveira 1989)

##### Notes

Freshwater (lake)

#### Anabaena inaequalis

Bornet & Flahault, 1886

1FAF86B2-90A8-535C-A933-4D1A13F04C1B

##### Distribution

Flores (Luz et al. 2020), Pico (Luz et al. 2020), São Miguel (Luz et al. 2020)

##### Notes

Freshwater (lake)

#### Anabaena torulosa

Lagerheim ex Bornet & Flahault, 1886

C1B36671-29CE-56F0-8A6A-081ECE6D1BBF

##### Distribution

Corvo (Trelease 1897)

##### Notes

Freshwater

#### Anabaenopsis

V.V.Miller, 1923

B645D965-F87A-5BF0-A39E-AEEF5BA728D8

##### Distribution

Corvo (Cordeiro et al. 2020b), Flores (Luz et al. 2020)

##### Notes

Freshwater (lake)

#### Anabaenopsis circularis

(G.S.West) Woloszynska & V.Miller in V.Miller, 1923

6B3731E2-A5FF-5165-9ECF-1BEBC72481B2

##### Distribution

Flores (Gonçalves 2008)

##### Notes

Freshwater (lake)

#### Anagnostidinema amphibium

(C.Agardh ex Gomont) Strunecký, Bohunická, J.R.Johansen & J.Komárek, 2017

3346370C-7D38-5534-A00C-B9D4226E09EB

##### Distribution

Flores (Bourrelly and Manguin 1946), São Miguel (Cedercreutz 1941), Terceira (Cedercreutz 1941)

##### Notes

Brackish (lake), freshwater (lake)

#### Anathece clathrata

(W.West & G.S.West) Komárek, Kastovsky & Jezberová, 2011

D865FE01-3303-5AB1-8080-DB04348ACF89

##### Distribution

São Miguel (Santos and Santana 2004)

##### Notes

Freshwater (lake)

#### Anathece minutissima

(West) Komárek, Kastovsky & Jezberová, 2011

CB0697C9-5FF4-5FD6-B3AB-DE2875CED4A4

##### Distribution

São Miguel (Luz et al. 2020)

##### Notes

Freshwater (lake)

#### Aphanizomenon

A.Morren ex É.Bornet & C.Flahault, 1886

F2B889F6-8C7A-5CC4-9D65-27039A0847C5

##### Distribution

São Miguel (Santos et al. 2001)

##### Notes

Freshwater (lake)

#### Aphanizomenon flos-aquae

Ralfs ex Bornet & Flahault, 1886

746F96B3-B418-564F-A851-BC6EC5320709

##### Distribution

Corvo (INOVA 1996), Flores (INOVA 1996), Graciosa (Azevedo et al. 2005), Pico (INOVA 1996), São Miguel (Krieger 1931)

##### Notes

Freshwater (lake)

#### Aphanizomenon gracile

Lemmermann, 1907

B4275013-C555-5491-A49B-7FA2E31BA156

##### Distribution

Flores (Luz et al. 2020), Pico (Luz et al. 2020), São Miguel (Luz et al. 2020)

##### Notes

Freshwater (lake)

#### Aphanizomenon manguinii

Bourrelly in Bourrelly & Manguin, 1952

8EBDF4DD-1806-5534-8691-5E77650B43E5

##### Distribution

Pico (Luz 2018)

##### Notes

Freshwater (lake)

#### Aphanocapsa

Nägeli, 1849

1E05D72B-365C-5C3C-BFE2-9569B08DBC37

##### Distribution

São Miguel (Santos and Santana 2004)

##### Notes

Freshwater (lake)

#### Aphanocapsa delicatissima

West & G.S.West, 1912

87DBC12D-68F6-5815-AA71-6FD82440167F

##### Distribution

Pico (Santos and Santana 2009b), São Miguel (Santos and Santana 2004)

##### Notes

Freshwater (lake)

#### Aphanocapsa elachista

West & G.S.West, 1894

8FD0214C-D1F9-5BCC-B9E7-DCF30A00BFA1

##### Distribution

São Jorge (Cedercreutz 1941), São Miguel (Santos and Santana 2004)

##### Notes

Freshwater (lake), terrestrial

#### Aphanocapsa grevillei

(Berkeley) Rabenhorst, 1865

39825678-DC02-5F4D-AAE9-7A5D6C688C1D

##### Distribution

São Jorge (Johansson 1977)

##### Notes

Freshwater

#### Aphanocapsa incerta

(Lemmermann) G.Cronberg & Komárek, 1994

54E2F8B0-827F-526B-863B-2652DB96E871

##### Distribution

São Miguel (Santos and Santana 2009a)

##### Notes

Freshwater (lake)

#### Aphanothece

Nägeli, 1849

BA39489C-B663-5554-A246-5D4BEC62B1EA

##### Distribution

Flores (Luz et al. 2020), São Miguel (Luz et al. 2020)

##### Notes

Freshwater (lake)

#### Aphanothece castagnei

(Kützing) Rabenhorst, 1865

497B852B-F6D3-5A40-9BF8-65C6038B0BFF

##### Distribution

Flores (Bourrelly and Manguin 1946)

##### Notes

Terrestrial

#### Aphanothece microscopica

Nägeli, 1849

E5C56523-31FD-5A31-91B1-3EF75A823619

##### Distribution

São Miguel (Bohlin 1901)

##### Notes

Freshwater (lake), terrestrial

#### Aphanothece naegelii

Wartmann in Rabenhorst, 1865

6DCFDD27-026B-589E-A429-349A6ED23A87

##### Distribution

São Miguel (Bohlin 1901)

##### Notes

Freshwater

#### Aphanothece nidulans

P.Richter, 1884

DC09DA52-E952-5BBD-BFCB-DB537B81C2A3

##### Distribution

São Miguel (Bourrelly and Manguin 1946)

##### Notes

Freshwater (lake)

#### Aphanothece pallida

(Kützing) Rabenhorst, 1863

2E7D654E-447C-5597-8F66-C5D97900A1C2

##### Distribution

São Miguel (Bourrelly and Manguin 1946)

##### Notes

Freshwater (lake)

#### Aphanothece saxicola

Nägeli, 1849

8411CDF5-C0CE-5678-BB2F-697D7C02E1E8

##### Distribution

São Miguel (Bohlin 1901)

##### Notes

Terrestrial

#### Aphanothece stagnina

(Sprengel) A.Braun in Rabenhorst, 1863

92387C66-DD74-547C-AC27-D22A52C37C46

##### Distribution

Pico (Johansson 1977)

##### Notes

Freshwater

#### Arthrospira

Sitzenberger ex Gomont, 1892

3D71333A-E7E2-5B03-B290-633181D35210

##### Distribution

São Miguel (Cordeiro et al. 2020b)

##### Notes

Freshwater (lake)

#### Calothrix

C.Agardh ex Bornet & Flahault, 1886

7EE8080E-33F4-5D0D-B13F-F56B3F7F90D5

##### Distribution

Flores (Cordeiro et al. 2020b), São Jorge (Luz 2018), Santa Maria (Cordeiro et al. 2020b), São Miguel (Cordeiro et al. 2020b)

##### Notes

Freshwater (lake, stream)

#### Calothrix breviarticulata

West & G.S.West, 1897

7F2A9670-72FA-5282-A8A1-657C0CB5CA08

##### Distribution

Flores (Luz 2018)

##### Notes

Freshwater (lake)

#### Calothrix castellii

Bornet & Flahault, 1886

EEB25B65-A92B-5B2A-A6A6-07FB71990651

##### Distribution

Pico (Luz 2018), São Miguel (Luz 2018)

##### Notes

Freshwater (lake)

#### Calothrix parietina

Thuret ex Bornet & Flahault, 1886

7421F633-1861-5DE5-99F0-3F466F984AC4

##### Distribution

São Miguel (Bohlin 1901)

##### Notes

Terrestrial

#### Chlorogloeopsis fritschii

(A.K.Mitra) A.K.Mitra & D.C.Pandey, 1967

61D91FBD-E047-5ACB-A99A-516EF09A6401

##### Distribution

São Miguel (Luz 2018)

##### Notes

Thermal (stream)

#### Chroococcus

Nägeli, 1849

6EA6AF4F-B38D-53ED-B0E8-4BAE0D8371AB

##### Distribution

São Miguel (Moseley 1874)

##### Notes

Freshwater (lake), thermal (spring)

#### Chroococcus dispersus

(Keissler) Lemmermann, 1904

E13877D8-7240-563C-961C-FB00B97DEBD4

##### Distribution

Pico (Santos and Santana 2009b), São Miguel (Santos and Santana 2009a)

##### Notes

Freshwater (lake)

#### Chroococcus membraninus

(Meneghini) Nägeli, 1849

D0F29FFA-1B90-5FF8-BEA8-6331700F7430

##### Distribution

São Miguel (Bohlin 1901)

##### Notes

Thermal (stream)

#### Chroococcus minor

(Kützing) Nägeli, 1849

6FCC9259-D6DF-5AC4-8A82-A2004AFACA42

##### Distribution

São Miguel (Archer 1874)

##### Notes

Freshwater (lake)

#### Chroococcus minutus

(Kützing) Nägeli, 1849

4F1AED92-AD9C-5A81-B4E8-4FC65E7E21A8

##### Distribution

Flores (Luz et al. 2020), Pico (Johansson 1977), São Miguel (Bourrelly and Manguin 1946)

##### Notes

Freshwater (lake)

#### Chroococcus tenax

(Kirchner) Hieronymus, 1892

C0A6C736-2475-581E-9B95-A841D97CBEA0

##### Distribution

São Miguel (Oliveira 1989)

##### Notes

Freshwater (lake)

#### Chroococcus turgidus

(Kützing) Nägeli, 1849

51399019-4C66-5844-9523-0BFE6A161DFC

##### Distribution

Corvo (Cedercreutz 1941), Faial (Johansson 1977), Flores (Cedercreutz 1941), Pico (Johansson 1977), São Jorge (Johansson 1977), São Miguel (Bohlin 1901), Terceira (Cedercreutz 1941)

##### Notes

Freshwater (lake), terrestrial

#### Chroococcus turicensis

(Nägeli) Hansgirg, 1887

F5B2773F-B33D-5A1B-802A-071C23288BE3

##### Distribution

Flores (Bourrelly and Manguin 1946)

##### Notes

Freshwater

#### Chroococcus westii

J.B.Petersen, 1923

45271513-58C3-51E7-BBD4-2E5F56FDCC4D

##### Distribution

São Miguel (Oliveira 1989)

##### Notes

Terrestrial

#### Coelosphaerium

Nägeli, 1849

327A108E-1D20-5FF7-A77F-34556379954C

##### Distribution

São Miguel (Santos et al. 2001)

##### Notes

Freshwater (lake)

#### Coelosphaerium kuetzingianum

Nägeli, 1849

732E25B0-EA89-59F3-8178-3D047DF4DF90

##### Distribution

São Miguel (Santos and Santana 2004)

##### Notes

Freshwater (lake)

#### Coleospermum

Kirchner ex Frank, 1886

ADD1E5D6-BA23-5557-8C20-25144AF9EADD

##### Distribution

Flores (Luz 2018), Pico (Luz 2018), São Miguel (Luz 2018)

##### Notes

Freshwater (lake), thermal (pool, spring)

#### Coleospermum goeppertianum

Kirchner ex Frank, 1886

9ACACF4D-5D2C-5DA9-B3E0-C89AF2C18D73

##### Distribution

São Miguel (Krieger 1931)

##### Notes

Freshwater (lake)

#### Cyanobacterium synechococcoides

Komárek 1999

4D270238-0B1E-5853-9CBB-2AFCEC985639

##### Distribution

São Miguel (Cordeiro et al. 2020b)

##### Notes

Freshwater (lake)

#### Cyanobium

R.Rippka & G.Cohen-Bazire, 1983

BC7204EE-5549-518E-B23E-F522E5A9E588

##### Distribution

São Miguel (Cordeiro et al. 2020b)

##### Notes

Freshwater (lake)

#### Cyanobium plancticum

(G.Drews, H.Prauser & D.Uhlmann) Komárek, J.Kopecký & Cepák, 1999

6510DFA6-A64B-5CAF-8366-EF20536A93DB

##### Distribution

São Miguel (Xavier et al. 2018)

##### Notes

Freshwater (lake)

#### Cyanosaccus

K.J.Lukas & S.Golubic, 1981

490DA872-E818-5B86-86C6-4DFE8D7740EC

##### Distribution

Faial (Wisshak et al. 2011)

##### Notes

Marine (intertidal)

#### Cylindrospermum

Kützing ex Bornet & Flahault, 1886

76D98B8A-1394-5587-B60D-DE3B1F3481A3

##### Distribution

Pico (Cedercreutz 1941), São Miguel (Cordeiro et al. 2020b)

##### Notes

Freshwater (lake, stream)

#### Cylindrospermum licheniforme

Kützing ex Bornet & Flahault, 1886

CD0072DE-A493-58B1-B413-2BF2DD2535A2

##### Distribution

São Miguel (Trelease 1897)

##### Notes

Freshwater

#### Cylindrospermum majus

Kützing ex Bornet & Flahault, 1886

CBB9E118-DC1A-5F6A-9EDF-A06F2BD09469

##### Distribution

Faial (Johansson 1977), Flores (Bourrelly and Manguin 1946), São Jorge (Johansson 1977), São Miguel (Trelease 1897)

##### Notes

Freshwater (lake)

#### Dichothrix baueriana

Bornet & Flahault, 1886

605545D7-3ADB-54CE-ACCB-E1ED74D5C226

##### Distribution

Corvo (Trelease 1897)

##### Notes

Freshwater

#### Dichothrix orsiniana var. africana

Frémy, 1924

4520628B-ECB1-566B-8BAA-9415AAF283FA

##### Distribution

Flores (Bourrelly and Manguin 1946)

##### Notes

Freshwater (lake)

#### Dolichospermum

(Ralfs ex Bornet & Flahault) P.Wacklin, L.Hoffmann & J.Komárek, 2009

C646515E-D412-5E81-A619-B70D9690425B

##### Distribution

São Miguel (Cordeiro et al. 2020b)

##### Notes

Freshwater (lake)

#### Dolichospermum affine

(Lemmermann) Wacklin, L.Hoffmann & Komárek, 2009

608983D2-24B1-5FB3-AEC7-518B93CCE7A8

##### Distribution

Pico (Santos and Santana 2009b), São Miguel (Oliveira 1989)

##### Notes

Freshwater (lake)

#### Dolichospermum circinale

(Rabenhorst ex Bornet & Flahault) Wacklin, L.Hoffmann & Komárek, 2009

B91AFB58-208C-5199-834C-428612019756

##### Distribution

São Miguel (Cordeiro 2015)

##### Notes

Freshwater (lake)

#### Dolichospermum delicatulum

(Lemmermann) Wacklin, L.Hoffmann & Komárek, 2009

DCB702F8-E4B4-51D9-9E3C-BB0BA69AA83F

##### Distribution

Corvo (Luz et al. 2020), Flores (Luz et al. 2020), Pico (Luz et al. 2020), São Miguel (Luz et al. 2020)

##### Notes

Freshwater (lake)

#### Dolichospermum flos-aquae

(Brébisson ex Bornet & Flahault) Wacklin, L.Hoffmann & Komárek, 2009

9AAD69CE-9507-5D0A-BF8D-4DCEEC859E4F

##### Distribution

São Miguel (INOVA 1996)

##### Notes

Freshwater (lake)

#### Dolichospermum planctonicum

(Brunnthaler) Wacklin, L.Hoffmann & Komárek, 2009

BD58B186-0E34-5D9C-AC6E-6456585EBFD1

##### Distribution

Pico (Luz et al. 2020), São Miguel (Luz et al. 2020)

##### Notes

Freshwater (lake)

#### Dolichospermum scheremetieviae

(Elenkin) Wacklin, L.Hoffmann & Komárek, 2009

1221A41B-8B2A-5882-83A8-EC38D2C68492

##### Distribution

Corvo (Luz et al. 2020), Flores (Luz et al. 2020), Pico (Santos and Santana 2009b), São Miguel (Oliveira 1989)

##### Notes

Freshwater (lake)

#### Dolichospermum sigmoideum

(Nygaard) Wacklin, L.Hoffmann & Komárek, 2009

D088D6B9-11F2-5244-BDE2-4DABC9EDEC1E

##### Distribution

Pico (Santos and Santana 2009b), São Miguel (Cordeiro 2015)

##### Notes

Freshwater (lake)

#### Dolichospermum solitarium

(Klebahn) Wacklin, L.Hoffmann & Komárek, 2009

E4A9E0C6-7AC7-5C97-A7A3-C9BF9608CE35

##### Distribution

Flores (Luz et al. 2020), Pico (Luz et al. 2020), São Miguel (Bourrelly and Manguin 1946), Terceira (Luz et al. 2020)

##### Notes

Freshwater (lake)

#### Dolichospermum spiroides

(Klebhan) Wacklin, L.Hoffmann & Komárek, 2009

5456697E-14D1-5872-9A51-463C92738403

##### Distribution

São Miguel (Luz et al. 2020)

##### Notes

Freshwater (lake)

#### Eucapsis alpina

F.E.Clements & H.L.Schantz, 1909

754423E1-3256-547E-B07F-C607CCC76555

##### Distribution

Corvo (Luz et al. 2020), Flores (Luz et al. 2020), Pico (Luz et al. 2020), São Miguel (Luz et al. 2020), Terceira (Luz et al. 2020)

##### Notes

Freshwater (lake)

#### Eucapsis minuta

F.E.Fritsch, 1912

CB370973-6823-52D0-97DC-D726C2358396

##### Distribution

São Miguel (Luz et al. 2020)

##### Notes

Freshwater (lake)

#### Fischerella

(Bornet & Flahault) Gomont, 1895

40F27740-A289-594B-909C-D13CFFACF8F2

##### Distribution

São Miguel (Cordeiro et al. 2020b)

##### Notes

Thermal (pool)

#### Fortiea

De Toni, 1936

D306D1CC-AAAE-5C46-B132-4F957B23002B

##### Distribution

Pico (Luz 2018)

##### Notes

Freshwater (lake)

#### Fortiea striatula

(F.C.Hy) De Toni, 1936

B6B3BC2F-0524-52FA-819A-1166708AEBF1

##### Distribution

Pico (Cordeiro et al. 2020b)

##### Notes

Freshwater (lake)

#### Geitlerinema ionicum

(Skuja) Anagnostidis, 1989

FC04E65F-29F9-5D3D-8396-2652EDB8D803

##### Distribution

Santa Maria (Bourrelly and Manguin 1946)

##### Notes

Terrestrial

#### Geitlerinema splendidum

(Greville ex Gomont) Anagnostidis, 1989

8390719B-3AA3-5717-8F40-68E97BD9EC77

##### Distribution

Pico (Luz et al. 2020), São Miguel (Cedercreutz 1941)

##### Notes

Freshwater (lake)

#### Gloeocapsa

Kützing, 1843

A37A7C2F-E744-5BFF-BE9D-BB661590FE41

##### Distribution

São Miguel (Luz et al. 2020)

##### Notes

Freshwater (lake)

#### Gloeocapsa atrata

Kützing, 1843

7BE6EE57-67E8-514B-914F-859004B4C25D

##### Distribution

São Miguel (Cedercreutz 1941), Terceira (Johansson 1977)

##### Notes

Freshwater, terrestrial

#### Gloeocapsa caldariorum

Rabenhorst, 1865

D4BAF589-2FDC-5142-8E17-AFA299A8F4AB

##### Distribution

Terceira (Johansson 1977)

##### Notes

Freshwater

#### Gloeocapsa compacta

Kützing, 1847

30649D81-E503-5960-BC44-04FEA3407185

##### Distribution

Flores (Bourrelly and Manguin 1946)

##### Notes

Freshwater (lake)

#### Gloeocapsa gelatinosa

Kützing, 1843

D3D4627F-5A99-5CCE-AC62-8B448724F8E3

##### Distribution

São Jorge (Johansson 1977), Terceira (Johansson 1977)

##### Notes

Freshwater

#### Gloeocapsa quaternata

Kützing, 1846

CFEC0C70-C9A3-5820-9EF4-D4149633E7F7

##### Distribution

São Miguel (Johansson 1977)

##### Notes

Freshwater

#### Gloeocapsa rupestris

Kützing, 1847

98995BAA-76E2-5DD9-97FE-2A19E17DB7FC

##### Distribution

São Jorge (Johansson 1977), Terceira (Johansson 1977)

##### Notes

Freshwater

#### Gloeocapsa thermalis

Kützing, 1843

C4DB2CC3-FED3-5ED7-A966-F96D11B9E93D

##### Distribution

São Miguel (Johansson 1977)

##### Notes

Thermal

#### Gloeocapsopsis dvorakii

(Novácek) Komárek & Anagnostidis ex Komárek 1993

0D8C7E2E-A879-5D1F-A7BA-4C4A5ECAAC3E

##### Distribution

São Miguel (Cordeiro et al. 2020b)

##### Notes

Thermal (pool)

#### Gloeocapsopsis magma

(Brébisson) Komárek & Anagnostidis ex Komárek, 1993

DFC47F20-3A6B-553C-9036-7665DF7A6678

##### Distribution

São Miguel (Bohlin 1901)

##### Notes

Terrestrial

#### Gloeothece cystifera

(Hassall) Rabenhorst, 1865

A7C4A689-2C1C-57CB-AAC5-CBA2D7318CC6

##### Distribution

São Miguel (Bohlin 1901)

##### Notes

Terrestrial

#### Gloeothece rupestris

(Lyngbye) Bornet in Wittrock & Nordstedt, 1880

DC2E50FA-F6D1-5806-B78F-E6B8FDD54616

##### Distribution

Flores (Bourrelly and Manguin 1946), São Miguel (Cedercreutz 1941)

##### Notes

Freshwater (lake)

#### Gloeotrichia pisum

Thuret ex Bornet & Flahault, 1886

09E5ADCA-D25A-5C28-AFEC-37D61E8AA44D

##### Distribution

São Miguel (Bohlin 1901)

##### Notes

Terrestrial

#### Goleter apudmare

Miscoe & J.R.Johansen, 2016

E2CF2F03-F5E2-5849-A65E-A8F40B75C549

##### Distribution

Flores (Cordeiro et al. 2020b)

##### Notes

Freshwater (lake)

#### Gomphosphaeria

Kützing, 1836

349C4809-0663-5289-BBAB-6BDF0F1F9C77

##### Distribution

São Miguel (Santos et al. 2001)

##### Notes

Freshwater (lake)

#### Hapalosiphon hibernicus

West & G.S.West, 1896

**Figure 2.**
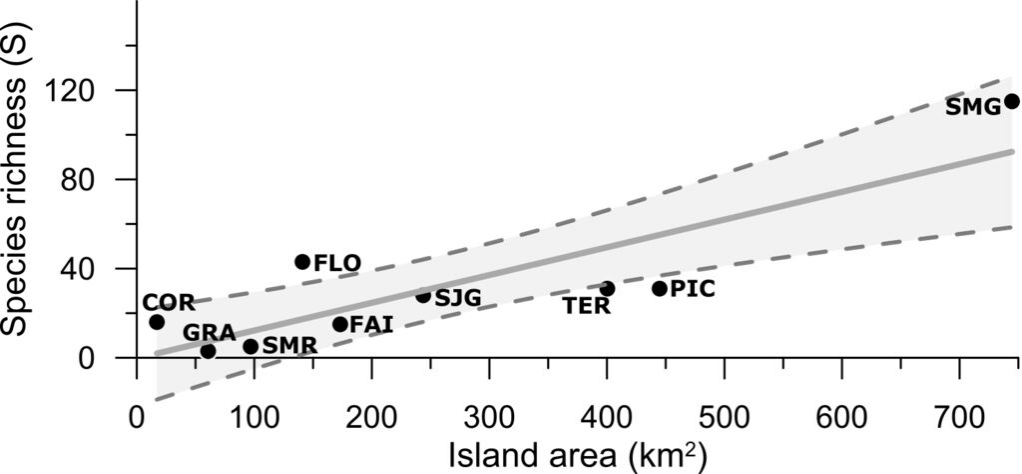
Species-area relationship. Regression line indicates a significant linear relationship with P = 0.003 and R^2^ = 0.86 (Pearson correlation). Dashed lines represent 95% interval confidence. COR: Corvo, FAI: Faial, FLO: Flores, GRA: Graciosa, PIC: Pico, SMR: Santa Maria, SJG: São Jorge, SMG: São Miguel, TER: Terceira.

C653B3E8-D5BE-54F8-ABB2-4ACB7EB84967

##### Distribution

Corvo (Cedercreutz 1941), Flores (Cedercreutz 1941), São Miguel (Bourrelly and Manguin 1946)

##### Notes

Freshwater (lake)

#### Hapalosiphon intricatus

West & G.S.West, 1894

32DF9264-1C08-5D22-A516-FC43697EDAFE

##### Distribution

São Jorge (Cedercreutz 1941), Terceira (Johansson 1977)

##### Notes

Freshwater (lake, stream)

#### Hapalosiphon pumilus

Kirchner ex Bornet & Flahault, 1887

47DFBC51-FC9C-56AB-9315-55CB3780F404

##### Distribution

Flores (Bourrelly and Manguin 1946), Santa Maria (Luz 2018)

##### Notes

Freshwater (stream), terrestrial

#### Hapalosiphon

Nägeli ex É.Bornet & C.Flahault, 1886

98E15AF8-C4EF-5A52-AC93-EB2168A2EB78

##### Distribution

São Miguel (Cordeiro et al. 2020b)

##### Notes

Terrestrial

#### Heteroleibleinia kuetzingii

(Schmidle) Compère, 1985

803EECD2-B18B-510A-89DE-7524AED43D2E

##### Distribution

São Jorge (Cedercreutz 1941)

##### Notes

Freshwater

#### Homoeothrix africana

G.S.West, 1912

C5EB2BAA-E51F-589A-B07F-B43890A79542

##### Distribution

São Jorge (Johansson 1977)

##### Notes

Freshwater

#### Hydrocoryne spongiosa

Schwabe ex Bornet & Flahault 1887

03DF7F50-C3A3-5112-94B5-AF68ADD1DAF0

##### Distribution

São Miguel (Cordeiro et al. 2020b)

##### Notes

Freshwater (lake)

#### Hyella

É.Bornet & C.Flahault, 1888

6588D41C-AF0E-5D30-BACF-2FFFB3C665AC

##### Distribution

Faial (Wisshak et al. 2011)

##### Notes

Marine (intertidal)

#### Hyella caespitosa

Bornet & Flahault, 1888

AFDB46F8-C22A-5062-BE0B-912F4391D908

##### Distribution

Faial (Wisshak et al. 2011)

##### Notes

Marine (intertidal)

#### Hyella gigas

Lukas & Golubic, 1983

85CAA134-6978-55EB-A7B6-BD600287FE89

##### Distribution

Faial (Wisshak et al. 2011)

##### Notes

Marine (intertidal)

#### Isocystis planctonica

Starmach 1962

92F4F0E2-A517-5298-8771-8F6BCA36A2B0

##### Distribution

Flores (Cordeiro et al. 2020b)

##### Notes

Freshwater (lake)

#### Kamptonema

O.Strunecký, J.Komárek & J.Smarda, 2014

8BED7D41-3DC7-5DA1-B982-E08CB245EEF8

##### Distribution

Flores (Cordeiro et al. 2020b)

##### Notes

Freshwater (lake)

#### Kamptonema formosum

(Bory ex Gomont) Strunecký, Komárek & J.Smarda, 2014

9939C7C1-000A-537D-9296-D8C2CFAA4EBE

##### Distribution

Santa Maria (Trelease 1897), São Miguel (Bohlin 1901), Terceira (Cedercreutz 1941)

##### Notes

Freshwater, brackish (lake), thermal (stream)

#### Kyrtuthrix dalmatica

Ercegovic, 1929

FC2C266E-CC53-529F-A8B7-E8A794B80998

##### Distribution

Faial (Wisshak et al. 2011)

##### Notes

Marine (intertidal)

#### Leptodesmis

Raabová, Kovacik & Strunecký, 2019

416BE2FB-5390-5BF0-A207-6DC44FBF4BCB

##### Distribution

São Miguel (Cordeiro et al. 2020b)

##### Notes

Freshwater (lake)

#### Leptolyngbya

Anagnostidis & Komárek, 1988

541015A2-AB83-51AD-81AC-793EA01FDF72

##### Distribution

São Jorge (Luz 2018), São Miguel (Luz 2018)

##### Notes

Freshwater (lake), marine (lake), thermal (pool, stream)

#### Leptolyngbya gelatinosa

(Woronichin) Anagnostidis & Komárek, 1988

4B830626-D8B2-53B7-8938-4135F4D35596

##### Distribution

São Miguel (Luz 2018)

##### Notes

Thermal (stream)

#### Leptolyngbya granulifera

(J.J.Copeland) Anagnostidis 1936

C2EBE813-7B38-5D80-B1C1-703CFBD03695

##### Distribution

São Miguel (Cordeiro et al. 2020b)

##### Notes

Thermal (pool, spring)

#### Leptolyngbya laminosa

(Gomont ex Gomont) Anagnostidis & Komárek, 1988

4E808AF6-FFC4-5188-85E6-FA70E21E70C7

##### Distribution

São Miguel (Trelease 1897)

##### Notes

Thermal (spring)

#### Leptolyngbya nostocorum

(Bornet ex Gomont) Anagnostidis & Komárek, 1988

81E64AEC-EA8C-56D1-B716-42ABE80F86D4

##### Distribution

São Miguel (Bohlin 1901)

##### Notes

Freshwater (lake)

#### Leptolyngbya ochracea

(Thuret ex Gomont) Anagnostidis & Komárek, 1988

FA3EA5D5-6F68-5FD1-ACEA-21B97685FB6B

##### Distribution

São Miguel (Bohlin 1901)

##### Notes

Thermal (pool)

#### Leptolyngbya rivulariarum

(Gomont) Anagnostidis & Komárek, 1988

AFDF8AF7-C5A4-5379-89A8-27FED5A8133B

##### Distribution

São Miguel (Bohlin 1901)

##### Notes

Freshwater (lake)

#### Leptolyngbya subuliformis

(Gomont) Anagnostidis 2001

039FE232-94C8-5E4F-833C-5DCE69EB496A

##### Distribution

São Miguel (Cordeiro et al. 2020b)

##### Notes

Thermal (spring)

#### Leptolyngbya valderiana

(Gomont) Anagnostidis & Komárek, 1988

42D201A5-4778-5DD8-AFD1-2BDDB89BD077

##### Distribution

São Miguel (Bourrelly and Manguin 1946)

##### Notes

Terrestrial

#### Limnothrix

M.-E.Meffert, 1988

01B69F67-4BB1-583A-979C-6098D2CFA6F1

##### Distribution

Flores (Cordeiro et al. 2020b), São Miguel (Cordeiro et al. 2020b)

##### Notes

Freshwater (lake)

#### Limnothrix planctonica

(Woloszynska) Meffert, 1988

422E794B-09DC-5C26-8098-9E60113DFD28

##### Distribution

São Miguel (Santos and Santana 2009a)

##### Notes

Freshwater (lake)

#### Lyngbya

C.Agardh ex Gomont, 1892

1394AE00-73BF-5720-9E8D-4C50AB36D3C5

##### Distribution

São Miguel (Santos and Santana 2004)

##### Notes

Freshwater (lake)

#### Lyngbya lutea

Gomont ex Gomont, 1892

F36215C1-C130-51A9-BF4A-56A2B165F739

##### Distribution

Terceira (Neto et al. 2009)

##### Notes

Marine

#### Lyngbya martensiana

Meneghini ex Gomont, 1892

B2589F32-A84E-5CAE-8803-BA40FA5DB33F

##### Distribution

São Miguel (Bohlin 1901), Terceira (Johansson 1977)

##### Notes

Freshwater, thermal, terrestrial

#### Mastigocladus

Cohn ex Kirchner, 1898

ADD76FAA-B7B9-51CC-AC79-70D1CA76489E

##### Distribution

São Miguel (Cordeiro et al. 2020b)

##### Notes

Thermal (spring, stream)

#### Mastigocladus laminosus

Cohn ex Kirchner, 1898

DC1E7734-3205-5857-BE27-D9A58E52D9CC

##### Distribution

São Miguel (Bohlin 1901)

##### Notes

Thermal (spring, pool, stream)

#### Mastigocoleus testarum

Lagerheim ex Bornet & Flahault, 1886

7D733C69-72DF-58F1-8E82-624DD0DAEBC8

##### Distribution

Faial (Wisshak et al. 2011)

##### Notes

Marine (intertidal)

#### Merismopedia

Meyen, 1839

3664D887-87A5-5ACF-8D81-350E4491F435

##### Distribution

São Miguel (Archer 1874), Pico (Luz et al. 2020)

##### Notes

Freshwater (lake)

#### Merismopedia glauca

(Ehrenberg) Kützing, 1845

06B94FB9-EA53-5880-A2EC-11F2DF98BB7A

##### Distribution

São Miguel (Cedercreutz 1941), Terceira (Cedercreutz 1941)

##### Notes

Freshwater (lake)

#### Merismopedia tenuissima

Lemmermann, 1898

8D51F50A-BD13-5ED3-92DA-76C84678A48D

##### Distribution

Graciosa (Azevedo et al. 2005), São Miguel (Oliveira 1989), Terceira (Luz et al. 2020)

##### Notes

Freshwater (lake)

#### Microchaete bulbosa

J.Copeland, 1936

CEA81422-B88D-507A-964C-D6F4BF0C8342

##### Distribution

São Miguel (Luz 2018)

##### Notes

Thermal (spring)

#### Microchaete tenera

Thuret ex Bornet & Flahault, 1886

1C3B2104-118D-5F9F-8BF2-DF992186E19D

##### Distribution

Pico (Luz 2018), São Miguel (Bohlin 1901)

##### Notes

Freshwater (lake), terrestrial

#### Microcoleus amoenus

(Gomont) Strunecky, Komárek & J.R.Johansen, 2013

8EB10850-E428-50B4-97E0-31EA76A3BCDD

##### Distribution

Flores (Bourrelly and Manguin 1946), São Miguel (Cedercreutz 1941)

##### Notes

Freshwater (lake)

#### Microcoleus autumnalis

(Gomont) Strunecky, Komárek & J.R.Johansen, 2013

1BD74790-908A-5C96-8B7E-AEA77CCD7732

##### Distribution

Flores (Cedercreutz 1941), São Miguel (Cedercreutz 1941)

##### Notes

Freshwater (stream), terrestrial

#### Microcoleus lyngbyaceus

Kützing ex Forti, 1907

2D1AAA68-E16F-54D3-A17F-5CAE600F154A

##### Distribution

Terceira (Neto et al. 2009)

##### Notes

Marine

#### Microcystis

Lemmermann, 1907

D0BD10AF-5680-54F2-8D50-79431D9E342A

##### Distribution

São Miguel (INOVA 1996)

##### Notes

Freshwater (lake)

#### Microcystis aeruginosa

(Kützing) Kützing, 1846

2DEBF0D2-749E-5B17-8DA6-A07B3DCE594B

##### Distribution

Corvo (Luz et al. 2020), Flores (INOVA 1996), São Miguel (Vasconcelos et al. 1994)

##### Notes

Freshwater (lake)

#### Microcystis flos-aquae

(Wittrock) Kirchner, 1898

861390ED-F70D-574A-A12D-8469593AC2BF

##### Distribution

Flores (Luz et al. 2020), São Miguel (Oliveira 1989)

##### Notes

Freshwater (lake)

#### Microcystis pulverea

(H.C.Wood) Forti, 1907

C73B7D99-7A17-5D65-AE82-8E4E3B389155

##### Distribution

Pico (Santos and Santana 2009b), São Miguel (Santos and Santana 2004)

##### Notes

Freshwater (lake)

#### Microcystis robusta

(H.W.Clark) Nygaard, 1925

A32BC5F3-66D5-54FC-87A8-41A428764EB6

##### Distribution

São Miguel (Santos and Santana 2009a), Terceira (Santos and Santana 2009b)

##### Notes

Freshwater (lake)

#### Nodularia harveyana

Thuret ex Bornet & Flahault, 1886

C9A5F77F-12BC-5F0D-8FA2-9846A59B9BF1

##### Distribution

Corvo (Trelease 1897), Terceira (Cedercreutz 1941)

##### Notes

Brackish (lake), marine

#### Nostoc

Vaucher ex Bornet & Flahault, 1886

0D64A689-B759-5FD5-8F43-E84FDC561634

##### Distribution

Corvo (Cedercreutz 1941), Flores (Cedercreutz 1941), Santa Maria (Bourrelly and Manguin 1946), São Miguel (Moseley 1874)

##### Notes

Freshwater (lake, stream), terrestrial

#### Nostoc carneum

C.Agardh ex Bornet & Flahault, 1886

3504816D-643E-5E8A-BC52-BA57396FBA60

##### Distribution

Faial (Johansson 1977)

##### Notes

Freshwater

#### Nostoc commune

Vaucher ex Bornet & Flahault, 1886

2ABBBB94-EA1F-5222-8F7C-6CE439EE2578

##### Distribution

Pico (Johansson 1977)

##### Notes

Freshwater (lake)

#### Nostoc ellipsosporum

Rabenhorst ex Bornet & Flahault, 1886

FE19FC1D-AD73-5DE3-B0DE-5B54BC182A77

##### Distribution

Corvo (Trelease 1897), São Miguel (Bohlin 1901)

##### Notes

Freshwater

#### Nostoc paludosum

Kützing ex Bornet & Flahault, 1886

EA708117-914D-55F6-B68B-0AA4D4836EA2

##### Distribution

Corvo (Cordeiro et al. 2020b), Santa Maria (Bourrelly and Manguin 1946), São Miguel (Bohlin 1901)

##### Notes

Freshwater (lake)

#### Nostoc punctiforme

Hariot, 1891

E1749B75-D37F-5E43-9D4F-764E93EA3339

##### Distribution

São Miguel (Bohlin 1901)

##### Notes

Freshwater (lake), terrestrial

#### Nostoc sphaericum

Vaucher ex Bornet & Flahault, 1886

C7A90830-003D-5439-BA48-09318FE82DB4

##### Distribution

Faial (Johansson 1977), Flores (Bourrelly and Manguin 1946), São Jorge (Johansson 1977), São Miguel (Bourrelly and Manguin 1946)

##### Notes

Freshwater (lake), terrestrial

#### Nostoc sphaeroides

Kützing ex Bornet & Flahault, 1886

BF7A8666-B84A-5336-9C50-A8562CE303E7

##### Distribution

Faial (Johansson 1977), São Jorge (Johansson 1977)

##### Notes

Freshwater

#### Nostoc verrucosum

Vaucher ex Bornet & Flahault, 1886

9081A85F-45F8-52FF-8A28-2CE46A1BB116

##### Distribution

São Miguel (Cedercreutz 1941), Terceira (Trelease 1897)

##### Notes

Freshwater (stream)

#### Nostochopsis lobatus

H.C.Wood ex Bornet & Flahault, 1886

8A5D8CAA-A884-5E5E-8181-EDD72490185E

##### Distribution

São Miguel (Bohlin 1901)

##### Notes

Terrestrial

#### Oscillatoria

Vaucher ex Gomont, 1892

DCBF69E6-84DD-51C8-BD2F-AD4D08A29F46

##### Distribution

Flores (Luz et al. 2020), Pico (INOVA 1996), São Miguel (Moseley 1874), Terceira (Neto et al. 2009)

##### Notes

Freshwater (lake), marine

#### Oscillatoria geitleri f. major

Bourrelly in Bourrelly & Manguin, 1946

624C3A67-32CB-5903-9830-A1F1B72051A6

##### Distribution

Flores (Bourrelly and Manguin 1946)

##### Notes

Freshwater (stream)

#### Oscillatoria planctonica

Woloszynska, 1912

F448449E-96CF-5022-AB0C-4B4696B4C372

##### Distribution

São Miguel (Oliveira 1989)

##### Notes

Freshwater (lake)

#### Oscillatoria princeps

Vaucher ex Gomont, 1892

DC340E66-4A89-5F9A-8D52-AAE724E171D7

##### Distribution

Terceira (Neto et al. 2009)

##### Notes

Marine

#### Oscillatoria sancta

Kützing ex Gomont, 1892

0EBB4815-E988-5846-AE6D-F85788925D50

##### Distribution

São Miguel (Bohlin 1901)

##### Notes

Terrestrial

#### Oscillatoria tenuis

C.Agardh ex Gomont, 1892

50EEEE63-B5C9-5190-8602-C0D3B894A7D4

##### Distribution

Corvo (Cedercreutz 1941), Faial (Cedercreutz 1941), Flores (Cedercreutz 1941), Graciosa (Cedercreutz 1941), Pico (Luz et al. 2020), São Miguel (Bohlin 1901), Terceira (Luz et al. 2020)

##### Notes

Freshwater (lake, stream), terrestrial

#### Pegethrix

Mai, J.R.Johansen & Bohunická, 2018

A2B01D08-5109-5AAA-8195-C13EA19C31A2

##### Distribution

São Miguel (Cordeiro et al. 2020b)

##### Notes

Freshwater (lake)

#### Petalonema velutinum

Migula, 1907

1FDA5E34-D314-5044-A091-E398FB8682D1

##### Distribution

Flores (Cedercreutz 1941)

##### Notes

Freshwater

#### Phormidium

Kützing ex Gomont, 1892

6D106293-F8FA-5AD2-9EAA-0BCA8DCCBF7E

##### Distribution

Corvo (Luz et al. 2020), Faial (Cedercreutz 1941), Pico (Luz et al. 2020), São Jorge (Luz 2018), São Miguel (Fish and Codd 1994)

##### Notes

Freshwater (lake), marine (lake), thermal

#### Phormidium aerugineo-caeruleum

(Gomont) Anagnostidis & Komárek, 1988

170CD658-E933-5F78-8EED-5BBDA9A07FC5

##### Distribution

São Miguel (Bohlin 1901)

##### Notes

Freshwater (lake)

#### Phormidium allorgei

(Frémy) Anagnostidis & Komárek, 1988

8745AAB7-6F51-581A-86C6-791A2403BC9B

##### Distribution

Terceira (Johansson 1977)

##### Notes

Freshwater

#### Phormidium breve

(Kützing ex Gomont) Anagnostidis & Komárek, 1988

F1939C00-6610-5FCC-993F-E00C5F8BA537

##### Distribution

São Miguel (Bohlin 1901)

##### Notes

Marine, thermal

#### Phormidium durum

N.L.Gardner, 1927

976B2EF6-DAE6-5169-AC4B-D2F22ACA0425

##### Distribution

São Jorge (Johansson 1977)

##### Notes

Freshwater

#### Phormidium irriguum

(Kützing ex Gomont) Anagnostidis & Komárek, 1988

F1217E6A-93E8-56C4-BD3A-35B2E2702A22

##### Distribution

São Jorge (Johansson 1977), Terceira (Bohlin 1901)

##### Notes

Freshwater (stream)

#### Phormidium pachydermaticum

Frémy, 1930

542492AB-F5B9-571F-8D6F-77150A2458DD

##### Distribution

São Jorge (Johansson 1977), Terceira (Johansson 1977)

##### Notes

Freshwater

#### Phormidium retzii

Kützing ex Gomont, 1892

5F2C1CBB-3DF1-5E29-9F40-213BE887C7D8

##### Distribution

Faial (Johansson 1977), São Jorge (Johansson 1977), São Miguel (Cedercreutz 1941)

##### Notes

Freshwater

#### Phormidium rotheanum

Itzigsohn in Rabenhorst, 1865

EEEAF295-3AEF-5C6F-8EA4-5ECF20493ED5

##### Distribution

São Jorge (Johansson 1977)

##### Notes

Freshwater

#### Phormidium terebriforme

(C.Agardh ex Gomont) Anagnostidis & Komárek, 1988

91BE4B1D-9F31-5A08-824C-1BE87AA1C733

##### Distribution

São Miguel (Bohlin 1901)

##### Notes

Thermal

#### Planktolyngbya

Anagnostidis & Komárek, 1988

424EF739-A621-5534-B535-EF66951A6898

##### Distribution

Flores (Luz et al. 2020), Pico (Luz et al. 2020), São Miguel (Cordeiro 2015)

##### Notes

Freshwater (lake)

#### Planktolyngbya limnetica

(Lemmermann) Komárková-Legnerová & Cronberg, 1992

081964B8-8A08-5C9E-8267-DB5602DA1713

##### Distribution

Pico (Luz et al. 2020), São Miguel (Cedercreutz 1941)

##### Notes

Freshwater (lake)

#### Planktothrix agardhii

(Gomont) Anagnostidis & Komárek, 1988

39DA6936-415F-5ADD-A16A-3AB4B772982D

##### Distribution

São Miguel (Santos and Santana 2009a)

##### Notes

Freshwater (lake)

#### Plectonema endolithicum

Ercegovic, 1932

BECB3156-D1B4-57DF-80EF-A214C58D8FE2

##### Distribution

Faial (Wisshak et al. 2011)

##### Notes

Marine (intertidal)

#### Plectonema terebrans

Bornet & Flahault ex Gomont, 1892

6A2CAE44-AE45-5789-B5C1-9EBCB17D6BA1

##### Distribution

Faial (Wisshak et al. 2011)

##### Notes

Marine (intertidal)

#### Pseudanabaena

Lauterborn, 1915

D7A53FFE-E282-5537-890B-7A77C1F7165F

##### Distribution

Corvo (Luz et al. 2020), Flores (Luz et al. 2020), Pico (Luz et al. 2020), São Miguel (Santos et al. 2005)

##### Notes

Freshwater (lake)

#### Pseudanabaena catenata

Lauterborn, 1915

C84CFF8A-311B-5950-A45C-3DD3C2489636

##### Distribution

São Miguel (Oliveira 1989)

##### Notes

Freshwater (lake)

#### Pseudanabaena limnetica

(Lemmermann) Komárek, 1974

DDD3F482-CBB4-57C6-87EF-B67D916046CD

##### Distribution

Corvo (Luz et al. 2020), Flores (Luz et al. 2020), Pico (Luz et al. 2020), São Miguel (Luz et al. 2020)

##### Notes

Freshwater (lake)

#### Pseudanabaena minima

(G.S.An) Anagnostidis, 2001

37631284-F293-59FC-9CE5-C0729E4C9404

##### Distribution

Pico (Luz 2018)

##### Notes

Freshwater (lake)

#### Pseudanabaena mucicola

(Naumann & Huber-Pestalozzi) Schwabe, 1964

31E35EB1-8639-5C50-A0AC-553E39D0D570

##### Distribution

São Miguel (Santos and Santana 2004)

##### Notes

Freshwater (lake)

#### Pseudophormidium pauciramosum

(Anissimova) Anagnostidis, 2001

87385094-7C2F-5121-9349-7C77CA85F825

##### Distribution

Santa Maria (Luz 2018)

##### Notes

Brackish

#### Raphidiopsis curvata

F.E.Fritsch & M.F.Rich, 1930

3A722206-9AB1-5D6E-9A17-CF13C825CC19

##### Distribution

Corvo (INOVA 1996), Flores (INOVA 1996), São Miguel (INOVA 1996)

##### Notes

Freshwater (lake)

#### Rivularia

C.Agardh ex Bornet & Flahault, 1886

42B20208-B89E-5807-94D2-3FBA4452FD97

##### Distribution

São Miguel (Cordeiro et al. 2020b), Terceira (Neto et al. 2009)

##### Notes

Freshwater, marine

#### Rivularia biasolettiana

Meneghini ex Bornet & Flahault 1886

3160A454-4A04-59F0-AFEE-655A8B61CE3E

##### Distribution

São Miguel (Cordeiro et al. 2020b)

##### Notes

Freshwater (lake)

#### Rivularia bullata

Berkeley ex Bornet & Flahault, 1886

9D4D8EE8-D694-5307-8DC9-9F3728882850

##### Distribution

São Miguel (Trelease 1897)

##### Notes

Freshwater

#### Rivularia nitida

C.Agardh ex Bornet & Flahault, 1886

A46388FB-F8D7-5479-BB64-B958C5C201BA

##### Distribution

Flores (Trelease 1897)

##### Notes

Freshwater

#### Schizothrix

Kützing ex M.Gomont, 1892

31F968A0-515F-5248-B3BC-8D059332E39A

##### Distribution

Terceira (Johansson 1977)

##### Notes

Freshwater

#### Schizothrix cuspidata

(West & G.S.West) West & G.S.West, 1896

08E0E674-48CD-5B5C-9F5B-ACCBD9BB343C

##### Distribution

Faial (Bourrelly and Manguin 1946)

##### Notes

Terrestrial

#### Schizothrix fuscescens

Kutzing ex Gomont, 1892

047A30CD-2CFD-5872-A650-870B836EE18D

##### Distribution

Terceira (Johansson 1977)

##### Notes

Freshwater

#### Schizothrix lacustris

A.Braun ex Gomont, 1892

7A9DF08F-78C6-5FAA-83B7-65DEFCB50236

##### Distribution

São Jorge (Johansson 1977)

##### Notes

Freshwater

#### Schizothrix pallida

(Kützing ex Forti) Geitler, 1932

72497F8B-92AA-56AA-B265-6EAFFAABA2C0

##### Distribution

Terceira (Neto et al. 2009)

##### Notes

Marine

#### Schizothrix symplocoides

(N.L.Gardner) Geitler, 1932

FBABED4B-739C-50F5-A2AC-9272B7C92A36

##### Distribution

Terceira (Johansson 1977)

##### Notes

Freshwater

#### Schizothrix telephoroides

Gomont, 1890

6E3A16CB-DF26-5765-A68D-117120FDDF50

##### Distribution

Faial (Johansson 1977)

##### Notes

Freshwater

#### Schizothrix vaginata

Gomont, 1890

5DCA9FDD-7E6D-56BC-AD98-E97330CBDFC2

##### Distribution

São Jorge (Johansson 1977)

##### Notes

Freshwater

#### Scytonema

C.Agardh ex É.Bornet & C.Flahault, 1886

70A0C04A-3976-5165-8064-FD9C492C6892

##### Distribution

Flores (Cedercreutz 1941)

##### Notes

Terrestrial

#### Scytonema amplum

West & G.S.West, 1895

299294E7-25B1-5DD5-8C14-8E0E2B7418CD

##### Distribution

São Miguel (Bourrelly and Manguin 1946)

##### Notes

Terrestrial

#### Scytonema dilatatum

Bharadwaja, 1934

3FC47D9F-74F5-501F-93D2-16148B94C535

##### Distribution

Terceira (Johansson 1977)

##### Notes

Freshwater

#### Scytonema guyanense

Bornet & Flahault, 1888

4D41D234-53B5-582B-BB8F-5AE76A05ABDE

##### Distribution

Flores (Bourrelly and Manguin 1946), São Miguel (Bourrelly and Manguin 1946)

##### Notes

Terrestrial

#### Scytonema hofmannii

C.Agardh ex Bornet & Flahault, 1886

EE7ACCD6-0733-5846-A65D-84F072549D31

##### Distribution

São Jorge (Johansson 1977), São Miguel (Cedercreutz 1941)

##### Notes

Freshwater (lake)

#### Scytonema javanicum

Bornet ex Bornet & Flahault, 1886

82B473F8-3801-57BC-8C6B-80DB19B69118

##### Distribution

Flores (Bourrelly and Manguin 1946)

##### Notes

Freshwater (lake)

#### Scytonema mirabile

Bornet, 1889

25239CE2-7AFC-5963-85D4-408D7A91CDEA

##### Distribution

Pico (Cedercreutz 1941), São Jorge (Cedercreutz 1941), São Miguel (Cedercreutz 1941), Terceira (Cedercreutz 1941)

##### Notes

Freshwater (lake, stream), terrestrial

#### Scytonema stuposum

Bornet ex Bornet & Flahault, 1887

64736D9B-8EFE-53DB-9CD5-BD27C912F2C4

##### Distribution

Flores (Bourrelly and Manguin 1946)

##### Notes

Freshwater (lake)

#### Scytonematopsis

E.I.Kiseleva, 1930

10ABBF2D-A791-5A8C-8518-8A784FEC0455

##### Distribution

Flores (Cordeiro et al. 2020b), Pico (Cordeiro et al. 2020b)

##### Notes

Freshwater (lake)

#### Snowella

A.A.Elenkin, 1938

DBE1C8D2-C455-537E-9828-C3AB301CE9DC

##### Distribution

São Miguel (Luz et al. 2020)

##### Notes

Freshwater (lake)

#### Snowella lacustris

(Chodat) Komárek & Hindák, 1988

63FC5AEE-E9C9-55CA-94C0-E9C24E7C50A7

##### Distribution

São Miguel (Santos and Santana 2004)

##### Notes

Freshwater (lake)

#### Sphaerospermopsis

Zapomelová, Jezberová, Hrouzek, Hisem, Reháková & Komárková, 2010

137EBCE7-E44A-5419-A1D3-ADF0E71501D8

##### Distribution

Pico (Luz 2018)

##### Notes

Freshwater (lake)

#### Sphaerospermopsis aphanizomenoides

(Forti) Zapomelová, Jezberová, Hrouzek, Hisem, Reháková & Komárková, 2010

65F558B6-ED44-5180-B435-CF311BE91388

##### Distribution

Pico (Santos and Santana 2009b)

##### Notes

Freshwater (lake)

#### Spirulina subsalsa

Oersted ex Gomont, 1892

3EE7C9AC-5043-5CC6-9045-6AE70547876C

##### Distribution

São Jorge (Luz 2018)

##### Notes

Marine (lake)

#### Stenomitos

Miscoe & J.R.Johansen, 2016

B32D9844-A013-54F3-B033-E57ED65D674D

##### Distribution

Pico (Cordeiro et al. 2020b)

##### Notes

Freshwater (lake)

#### Stigonema hormoides

Bornet & Flahault, 1886

6BD919A7-E6FF-5A3A-B580-1D0D03ADD80F

##### Distribution

Flores (Bourrelly and Manguin 1946), São Jorge (Johansson 1977), São Miguel (Cedercreutz 1941), Terceira (Cedercreutz 1941)

##### Notes

Freshwater (lake), terrestrial

#### Stigonema informe

Kützing ex Bornet & Flahault, 1886

6F5FAA7E-D4FE-5EC1-A7E6-8418FB59DE6C

##### Distribution

São Jorge (Johansson 1977), Terceira (Johansson 1977)

##### Notes

Freshwater

#### Stigonema mamillosum

C.Agardh ex Bornet & Flahault, 1886

22169D47-4EEB-5C16-ABCD-76F02AB91E49

##### Distribution

São Jorge (Johansson 1977), São Miguel (Bourrelly and Manguin 1946)

##### Notes

Freshwater, terrestrial

#### Stigonema minutum

Hassall ex Bornet & Flahault, 1886

754A2D0E-CA33-5F36-8B5C-C44ED15B7BB0

##### Distribution

Flores (Bourrelly and Manguin 1946), São Jorge (Johansson 1977), São Miguel (Bohlin 1901), Terceira (Johansson 1977)

##### Notes

Freshwater (lake)

#### Stigonema multipartitum

N.L.Gardner, 1927

277B6A92-D8C9-512F-9388-6E4A5D9C0288

##### Distribution

São Jorge (Johansson 1977)

##### Notes

Freshwater

#### Stigonema ocellatum

Thuret ex Bornet & Flahault, 1886

B9F5BB81-4CFC-5CD5-8B5E-C85CD2BD0696

##### Distribution

Flores (Cedercreutz 1941), São Miguel (Cedercreutz 1941), Terceira (Johansson 1977)

##### Notes

Freshwater (lake)

#### Stigonema panniforme

Bornet & Flahault, 1886

1330F108-17FC-5C05-B141-A525244AD341

##### Distribution

São Miguel (Bourrelly and Manguin 1946)

##### Notes

Freshwater (stream)

#### Stigonema robustum

N.L.Gardner, 1927

8A74E630-C1AF-58C7-B0E9-22CBED7AACF1

##### Distribution

São Jorge (Johansson 1977)

##### Notes

Freshwater

#### Stigonema tomentosum

Hieronymus, 1895

1045DD00-12D4-53AA-825D-C66527124414

##### Distribution

São Jorge (Johansson 1977), São Miguel (Cedercreutz 1941)

##### Notes

Freshwater, terrestrial

#### Symploca dubia

Gomont, 1892

A43E25D7-0839-504E-80F4-D75E2F3E3FC9

##### Distribution

São Miguel (Cedercreutz 1941)

##### Notes

Thermal (pool)

#### Symploca thermalis

Gomont, 1892

2E16802F-02EF-5943-8166-25744AD2B127

##### Distribution

São Miguel (Bohlin 1901)

##### Notes

Thermal

#### Synechococcus

Nägeli, 1849

D0B2F7AA-BF3C-5370-8A8F-7CBAD85CAAF6

##### Distribution

São Miguel (Luz et al. 2020), Terceira (Luz et al. 2020)

##### Notes

Freshwater (lake)

#### Synechococcus nidulans

(Pringsheim) Komárek, 1970

C77F166C-9212-5512-A98A-58F52E052F9E

##### Distribution

São Miguel (Xavier et al. 2018)

##### Notes

Freshwater (lake)

#### Synechocystis

C.Sauvageau, 1892

056B3AAE-A756-5693-BE6B-956F18AA1215

##### Distribution

São Miguel (Luz et al. 2020), Pico (Luz et al. 2020)

##### Notes

Freshwater (lake)

#### Tildeniella torsiva

Mai, J.R.Johansen & Pietrasiak, 2018

9B23D44E-FAC3-501A-818D-EAB3F9F691E1

##### Distribution

São Miguel (Cordeiro et al. 2020b)

##### Notes

Freshwater (lake)

#### Tolypothrix

Kützing ex É.Bornet & C.Flahault, 1886

7520D80D-06F6-5E30-B71E-9004D6CB5EE0

##### Distribution

São Miguel (Archer 1874)

##### Notes

Freshwater

#### Tolypothrix distorta

Kützing ex Bornet & Flahault, 1886

2D14EF00-E006-5AE9-8B5F-C1CC1C660B3C

##### Distribution

São Miguel (Bohlin 1901)

##### Notes

Freshwater

#### Tolypothrix helicophila

Lemmermann, 1910

06F802E4-E334-50CB-AB11-E3583746C1BB

##### Distribution

Pico (Cordeiro et al. 2020b)

##### Notes

Freshwater (lake)

#### Tolypothrix lanata

Wartmann ex Bornet & Flahault, 1886

2DB9AB26-62EF-5163-9B99-0A2FEDD0EFAF

##### Distribution

São Miguel (Cedercreutz 1941)

##### Notes

Freshwater (lake)

#### Tolypothrix tenuis

Kützing ex Bornet & Flahault, 1886

0649CE44-C607-5C1E-A860-CA690555C2F8

##### Distribution

Flores (Bourrelly and Manguin 1946)

##### Notes

Freshwater

#### Trichormus variabilis

(Kützing ex Bornet & Flahault) Komárek & Anagnostidis, 1989

6CC32149-43D9-50EE-A5D7-E394851983EA

##### Distribution

São Miguel (Bohlin 1901)

##### Notes

Thermal (pool)

#### Tychonema

K.Anagnostidis & J.Komárek, 1988

E422F595-49ED-5FCA-98EB-F0AABA8BCE92

##### Distribution

São Miguel (Cordeiro et al. 2020b)

##### Notes

Freshwater (lake)

#### Westiellopsis

Janet, 1941

0ADAA7EE-9A01-588E-9516-525C8CC18002

##### Distribution

São Miguel (Cordeiro et al. 2020b)

##### Notes

Freshwater (lake), thermal (stream)

#### Woronichinia naegeliana

(Unger) Elenkin, 1933

4FBC75DF-F1C4-5FB8-B0A8-E83DA94C0A07

##### Distribution

Corvo Luz et al. 2020), Flores (Luz et al. 2020), Pico (Luz et al. 2020), São Miguel (Santos and Santana 2004)

##### Notes

Freshwater (lake)

## Analysis

The present work comprises 225 taxa, 179 identified species and 11 only to genus level, distributed by six orders (Chroococcales, Nostocales, Oscillatoriales, Pleurocapsales, Spirulinales and Synechococcales), 30 families and 79 genera (Table 4). Most species belong to the Nostocales (43.0%) and Synechococcales (21.2%) orders. Chroococcales and Oscillatoriales orders contributed almost with the same number of species (17.3% and 16.8%, respectively), despite their different genera contributions.

A summary of cyanobacteria species richness found in the Azores and on each of the nine islands in the different types of habitats is given in Table 5. The number of recorded species was highest on São Miguel Island (115) and lowest on Graciosa Island (3). Freshwater systems were the most diverse habitats, comprising 193 taxa (85.7%), followed by thermal, with 23 species (10.2%), marine (13 species, 5.8%) and brackish systems (3 species, 1.3%).

A positive Pearson correlation coefficient (r = 0.86, n = 9, P = 0.003) was evident between species richness (S) and island area. This correlation is best described by a linear relationship (Fig. 2), where Pico and Flores seem to be outliers. Flores presented higher, while Pico has lower than expected species richness concerning its surface area.

## Discussion

**Figure 3.**
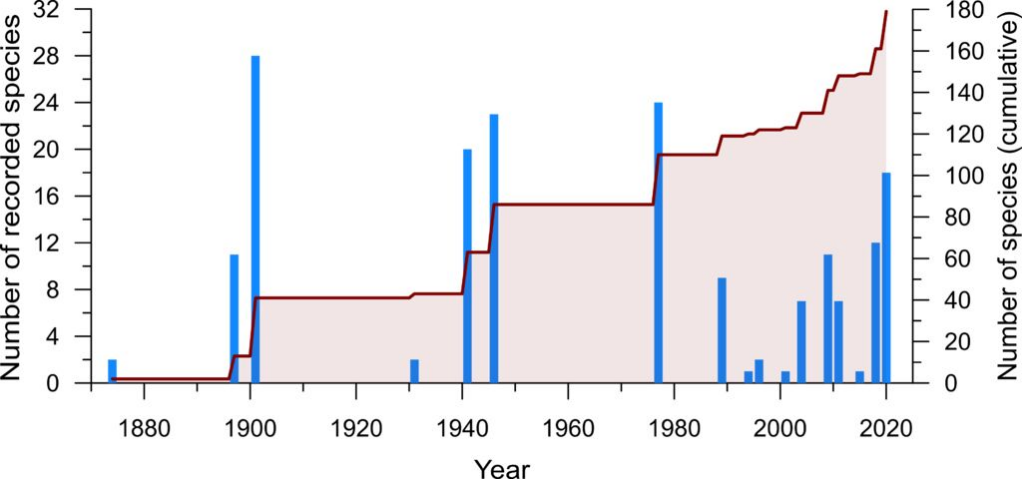
The number of described species through time from 1874 to 2020. Bars represent the number of species described per year, the line represents the cumulative number of described species.

The cyanobacteria diversity in the Azores Archipelago is understudied compared to other European regions (Gkelis et al. 2016), despite being one of the best studied archipelagos in the North Atlantic (Cordeiro et al. 2020a). With its first records of cyanobacteria in Furnas, São Miguel Island, under the Challenger expedition, Moseley (1874) identified three genera: *Chroococcus* Nägeli, 1849, *Nostoc* Vaucher ex Bornet & Flahault, 1886 and *Oscillatoria* Vaucher ex Gomont, 1892. After that, as seen in Fig. 3, the contributions of the Cyanobacteria flora were sporadic, but significant, mainly with Bohlin (1901), Cedercreutz (1941), Bourrelly and Manguin (1946) and Johansson (1977). Their contributions were important, but geographically restricted to the larger islands, such as São Miguel and Terceira. From thereon, the number of recorded cyanobacteria species in the Azores has risen throughout the end of the 20^th^ century and the 21^st^ century and presently stands at 225 taxa with 179 identified species (Fig. 3). This boost in the 21^st^ century is mainly due to the implementation of the WFD (e.g. Santos et al. 2005, Santos et al. 2012, Luz et al. 2020) with 39 new described species, which makes 21.8% of the total described species. This programme has helped the continuous study of freshwater cyanobacteria present in the major lakes of the Azores, in Pico, Flores and São Miguel. The works of Luz (2018), Xavier et al. (2018) and Cordeiro et al. (2020b) significantly contributed to the records of cyanobacteria for the Azores, reporting 19 new species, 10.6% of all taxa recorded only through in-vitro cultivation methods. Using a cultivation approach, these studies were able to isolate strains from lakes, terrestrial and thermal habitats, increasing the ability to identify small or rare species normally not detected in regular monitoring works.

Freshwater cyanobacteria are the most represented taxa in the Azores records, mainly from lakes. Although this result may reflect the abundance of this type of habitat in the Azores, it may also denote the less effort on diversity studies in other types of habitats. A much lower percentage of cyanobacteria was identified from thermal, marine and brackish habitats (10%, 6% and 1%, respectively), probably due to low sampling efforts. The availability of freshwater habitats in the Azores favours the establishment of incoming cyanobacteria in São Miguel, Flores, Terceira and São Jorge. These Islands have permanent streams, lakes, peat bogs and wetlands, providing highly diverse habitats for incomers, while these are absent in Faial, Graciosa and Santa Maria.

Several islands of the Azores have active volcanoes and present high numbers of fumarolic fields, geysers and hot springs (Cruz and França 2006) creating conditions for the growth of thermophilic cyanobacteria. Nevertheless, the current knowledge about thermal cyanobacteria in the Azores is low with few published works focusing on this habitat (Moseley 1874, Bohlin 1901, Luz 2018, Cordeiro et al. 2020b). The recent works by Luz (2018), with morphological identifications and Cordeiro et al. (2020b), that used both morphological and genetic characters for its identification, contributed to several new cyanobacteria taxa reports for the Azores from thermal habitats in São Miguel.

The cyanobacteria diversity and distribution in lotic systems in the Azores are much less known compared to other sites (Branco et al. 2001, Casamatta and Hašler 2016). Only a few works are available addressing this type of habitat in the Azores, with contributions mainly by Cedercreutz (1941), Bourrelly and Manguin (1946) and Johansson (1977), with no relevant works in the latest years. This is unusual as, in lotic systems, cyanobacteria are easily identified and sometimes even the dominant taxa (Schultz et al. 2013, Casamatta and Hašler 2016).

One of the most accepted explanations for regional biodiversity is the species-area relationship (SAR), according to which the number of species along the spatial scale increases with the area (e.g. Rosenzweig 1995, Drakare et al. 2006). This pattern was thoroughly studied on islands (e.g. Lomolino and Weiser 2001, Whittaker and Fernandez-Palacios 2007, Triantis et al. 2012), where the number of species from different taxonomic groups increases with the increase in island size (Triantis et al. 2012). In our data, a positive relationship between the island area and the number of species was observed (Fig. 3). This increase in cyanobacteria species richness with increasing island area in the Azores is consistent with the work of Borges et al. (2005), for arthropods and bryophytes and Raposeiro et al. (2009) for chironomids with an exception from Flores and Corvo Islands. A possible explanation for these exceptions is the higher percentage that water bodies represent in the total island area (Porteiro 2000) and also the higher precipitation (Secretaria Regional do Ambiente e do Mar 2011, Reichwaldt and Ghadouani 2012, Haakonsson et al. 2017). Compared to Pico Island, the percentage of land covered with water is double in Corvo and almost six times higher in Flores (Porteiro 2000). This suggests that, for cyanobacteria in the Azores, habitat diversity is an important factor in determining the SAR, as shown for other taxonomic groups and islands (Hortal et al. 2009, Chase et al. 2019).

Compared to other North Atlantic islands, the Azores present the highest species richness (Table 6). The overall distribution of species richness in the different cyanobacteria orders on the North Atlantic archipelagos generally follows the same pattern as the total world species, with Nostocales and Oscillatoriales being the richest orders. However, Nostocales represents a much higher contribution to the regional species richness in the Azores and Madeira Archipelagos, which could reflect their longer dispersion capabilities (Ribeiro et al. 2018). Nostocacean cyanobacteria are able to produce akinetes that can resist long periods of unfavourable conditions (Sarma 2012), enabling them to survive during long dispersion routes and colonise remote oceanic archipelagos, such as the Azores and Madeira. The absence of some orders, such as the Chroococcidiopsidales and Thermostichales, in the Azores and the other Macaronesia Archipelagos, suggests that they probably have a more restricted geographic distribution. Although biological, geographical and climatic factors may contribute to cyanobacteria regional species richness (Moreira et al. 2013, Walter et al. 2017, Ribeiro et al. 2018), the differences amongst islands are most probably related to different sampling efforts (Cordeiro et al. 2020b) and, between Azorean Islands, the distribution of planktic cyanobacteria seems to be mainly related to lake typology rather than environmental parameters (Cordeiro et al. 2020c). For instance, the reduced richness of Oscillatoriales in the Azores could be related to their preference for terrestrial and benthic habitats that are less studied in this Archipelago. With the increase in sampling campaigns covering all types of habitats, the reports of new cyanobacteria in the Azores are expected to increase and the representation of the different orders can become similar to the global pattern.

The hereby presented taxonomic list of cyanobacteria in the Azores represents a valuable resource for biodiversity research and awareness of described cyanobacteria tracked through years that, in the future, will allow the identification of possible invader species and studies of the influence of temperature changes in the World. Besides that, knowing the biodiversity of a specific archipelago enriches its value and allows future works in ecology and, in a more practical way, in biotechnology or pharmaceutical if found to be of increased value.

## Supplementary Material

E7D010BD-12C2-5F24-942D-018708242006 10.3897/BDJ.10.e87638.suppl1 Supplementary material 1 Cyanobacteria checklist of the Azores Archipelago, Portugal Data type Darwin Core Archive (.zip) of cyanobacteria taxa and occurrence data used for the presented and analysed checklist. Brief description Published Darwin Core Archive with two data tables in GBIF (doi: 10.15468/bfktqo) about the reports of cyanobacteria in the Azores Archipelago. The taxon (core) data table contains 229 records of cyanobacteria (from class to species level). One extension data table exists, with a total of 2838 occurrence records of cyanobacteria found in literature. The taxon data table is constructed, based on the occurrence data table. File: oo_694835.zip https://binary.pensoft.net/file/694835 Luz R, Cordeiro R, Fonseca A, Gonçalves V

## Figures and Tables

**Table 1.** Island characterisation. [1] Ávila et al. 2016, [2] Secretaria Regional do Ambiente e do Mar 2011, [3] Porteiro 2000, [4] Morton 2014, [5] Cruz and França 2006, [6] Secretaria Regional da Agricultura e Ambiente – Direção Regional do Ambiente 2015, [7] Ramsar 2013, [8] Morton et al. 1997.

**Group**	**Island**	**Age (Ma)^[1]^**	**Area (Km^2^)^[2]^**	**Freshwater lakes (N)^[3]^**	**Permanent Streams (N)^[6]^**	**Coastal lakes (N)^[4, 7, 8]^**	**Thermal waters (N)^[5]^**	**Total lake area (km^2^)^[3, 4, 7, 8]^**	**Annual precipitation (mm)^[2]^**
**Eastern**	São Miguel	4.00	744,6	33	6	-	14	8.34	1027.1
	Santa Maria	6.30	96,9	-	1	-	-	-	775.2
**Central**	Terceira	0.40	400,3	18	-	3	-	0.36	1125.6
	Pico	0.27	444,8	28	-	-	-	0.16	956.3
	Faial	0.85	173,1	-	-	-	2	-	974.0
	São Jorge	1.32	243,7	-	-	2	-	0.86	1194.3
	Graciosa	0.70	60,7	-	-	-	1	-	918.4
**Western**	Flores	2.16	141,0	8	2	-	2	0.72	1716.1
	Corvo	1.50	17,1	1	-	-	-	0.24	1144.6

**Table 2.** Darwin Core terms used in the occurrence data table.

Column label	Column description
id	Identifier.
type	The nature of the resource.
collectionCode	Acronym identifying the collection from which the record was derived.
basisOfRecord	Specific nature of the data record.
occurrenceID	Occurrence identifier.
catalogNumber	Identifier for the record within the collection.
associatedReferences	Literature associated with the occurrence.
eventDate	Date-time or interval during which the event was recorded.
continent	Name of the continent in which the occurrence location occurs.
waterBody	Name of the water body in which the occurrence location occurs.
islandGroup	Name of the island group in which the occurrence location occurs.
island	Name of the island on which the occurrence location occurs.
country	Name of the country in which the occurrence location occurs.
countryCode	Standard code for the country in which the occurrence location occurs.
municipality	Name of the municipality in which the occurrence location occurs.
locality	Name of the locality in which the occurrence location occurs.
decimalLatitude	Geographic latitude in which the occurrence location occurs.
decimalLongitude	Geographic longitude in which the occurrence location occurs.
geodeticDatum	Geodetic datum upon which the geographic coordinates given are based.
taxonID	Taxon identifier.
scientificName	The full scientific name including author.
acceptedNameUsage	The full scientific name including author currently accepted.
kingdom	Kingdom name in which the taxon is classified.
phylum	Phylum name in which the taxon is classified.
class	Class name in which the taxon is classified.
taxonRank	Lowest taxonomic rank of the taxon.

**Table 3.** Darwin Core terms used in the taxon data table.

Column label	Column description
id	Identifier.
taxonID	Taxon identifier.
scientificName	The full scientific name including author.
kingdom	Kingdom name in which the taxon is classified.
phylum	Phylum name in which the taxon is classified.
class	Class name in which the taxon is classified.
order	Order name in which the taxon is classified
family	Family name in which the taxon is classified.
genus	Genus name in which the taxon is classified.
specificEpithet	Species epithet name in which the taxon is classified.
infraspecificEpithet	Infraspecific epithet name in which the taxon is classified.
taxonRank	Lowest taxonomic rank of the taxon.
scientificNameAuthorship	Authorship information for the scientific name.

**Table 4.** Cyanobacteria taxa richness of the Azores Archipelago.

**Order**	**Taxa**	**Family**	**Genus**	**Species**		**Habitat (by taxa)**			
				Nº	%	Freshwater	Thermal	Brackish	Marine
**Chroococcales**	36	5	8	31	17.3	34	2	-	-
**Nostocales**	95	11	34	77	43.0	85	9	1	4
**Oscillatoriales**	36	4	14	30	16.8	27	7	2	2
**Pleurocapsales**	4	1	2	2	1.1	-	-	-	4
**Spirulinales**	1	1	1	1	0.6	-	-	-	1
**Synechococcales**	53	8	20	38	21.2	47	5	-	2
**Total**:	225	30	79	179	100	193	23	3	13

**Table 5.** Cyanobacteria taxa richness in the Azores by island.

	**Island**	**Taxa**	**Taxonomy**				**Habitat**			
			Order	Family	Genus	Species	Freshwater	Thermal	Brackish	Marine
**Eastern**	São Miguel	151	4	25	59	115	133	20	1	1
	Santa Maria	7	2	5	6	5	5	-	2	-
**Central**	Terceira	37	4	14	21	31	28	1	2	4
	Pico	43	4	16	27	31	43	-	-	-
	São Jorge	31	5	14	15	28	28	-	-	3
	Graciosa	3	3	3	3	3	3	-	-	-
	Faial	18	5	8	11	15	10	-	-	8
**Western**	Flores	56	4	18	33	43	56	-	1	-
	Corvo	21	4	11	16	16	20	-	-	1

**Table 6.** Cyanobacteria species richness in the Azores compared to world-known species richness (World's order and species number retrieved from Guiry and Guiry (2022); Canary Islands, Madeira and Cuba numbers retrieved from Cordeiro et al. (2020a)).

**Order**	**Azores**		**Madeira**		**Canary Islands**		**Cuba**		**World**		
	Nº	%	Nº	%	Nº	%	Nº	%	Nº	%	
**Chroococcales**	31	17.32	2	8.33	4	6.35	28	19.05	649	13.20	
**Chroococcidiopsidales**	0	0	0	0	0	0	2	1.36	37	0.75	
**Gloeobacterales**	0	0	0	0	0	0	0	0	3	0.06	
**Gloeomargaritales**	0	0	0	0	0	0	0	0	1	0.02	
**Nostocales**	77	43.02	11	45.83	20	31.75	44	29.93	1547	31.46	
**Oscillatoriales**	30	16.76	8	33.33	18	28.57	30	20.41	1397	28.41	
**Pleurocapsales**	2	1.12	1	4.17	6	9.52	3	2.04	223	4.53	
**Spirulinales**	1	0.56	0	0	4	6.35	5	3.40	56	1.14	
**Synechococcales**	38	21.23	2	8.33	11	17.46	35	23.81	995	20.23	
**Thermostichales**	0	0	0	0	0	0	0	0	10	0.20	
**Total**:	179		24		63		147		4918		
